# Use of granulocyte colony-stimulating factor in patients with chemotherapy-induced neutropaenia

**DOI:** 10.4102/hsag.v28i0.2221

**Published:** 2023-03-31

**Authors:** Lucky L. Shokane, Selente Bezuidenhout, Maryke Lundie

**Affiliations:** 1 Department of Clinical Pharmacy, School of Pharmacy, Sefako Makgatho Health Sciences University, Pretoria, South Africa; 2 Department of Public Health Pharmacy and Management, School of Pharmacy, Sefako Makgatho Health Sciences University, Pretoria, South Africa; 3 Department of Pharmaceutical Sciences, School of Pharmacy, Sefako Makgatho Health Sciences University, Pretoria, South Africa

**Keywords:** chemotherapy, febrile neutropaenia, G-CSF, guidelines compliance, dosage

## Abstract

**Background:**

Febrile neutropaenia (FN) and resultant infections are the major cause of treatment-related morbidity and mortality in patients receiving chemotherapy. Clinical practice guidelines recommend the use of granulocyte colony-stimulating factors (G-CSF) to reduce the risk of FN and ensuing complications in patients receiving chemotherapy. Despite these recommendations, inappropriate usage of G-CSF has been reported.

**Aim:**

To assess prescribing patterns and adherence to international guidelines of G-CSF in adult patients with chemotherapy-induced neutropaenia (CIN) at the haematology oncology wards of the Dr George Mukhari Academic Hospital (DGMAH) and compliance to guidelines.

**Methods:**

Medical records of adult patients who received G-CSF were reviewed retrospectively between 01 January 2018 and 31 July 2018.

**Results:**

Of the 128 patient files screened, 57 cases met the inclusion criteria. Duration of treatment with G-CSF was not in accordance with guidelines in more than 50% of the patients and in 43.86%, G-CSF dosing deviated from recommended guidelines.

**Conclusion:**

The study demonstrated over-prescribing of G-CSF due to either increased doses or duration of G-CSF therapy. Although prescribed for the correct indication, the dosage was often too high or the duration was too long, even once an acceptable neutrophil nadir count was reached. Interventions to optimise the use of G-CSF are required and the pharmacist may play a role in this regard.

**Contribution:**

The administration of the correct doses of G-CSF can reduce both the severity and duration of neutropaenia. Over-prescribing and incorrect dosing may contribute to patient morbidity and add to the financial burden of healthcare.

## Introduction

Chemotherapy-induced neutropaenia (CIN) is a serious side effect of chemotherapy which may contribute to complications and treatment-related deaths in oncology patients (Osmani et al. 2017). Chemotherapy-related neutropaenia may limit or delay future cycles of chemotherapy treatment (Kasi & Grothey 2018). Delayed cycles and treatment interruptions may negatively impact the overall survival of patients with potentially curable malignancies.

Neutropaenic patients are at an increased risk of developing serious infections which can be life-threatening when left untreated (Hashiguchi et al. 2015). Febrile neutropaenia (FN), a major dose-limiting toxicity of myelosuppressive chemotherapy, is described by clinical practice guidelines as neutropaenia with a single oral or tympanic temperature greater than or equal to 38.3 °C or greater than or equal to 38 °C for at least 1 h (Lucas, Olin & Coleman 2018). Although guidelines and treatment protocols are available, 10% – 30% of patients develop severe complications and eventually demise (Sereeaphinan, Kanchanasuwan & Julamanee 2021).

The administration of granulocyte colony-stimulating factor (G-CSF) is recommended as primary prophylaxis in clinical practice guidelines to reduce the risk of FN, with a resultant decreased risk of complications in patients receiving chemotherapy (Laali et al. 2020). The prophylactic use of G-CSF reduces the occurrence of FN and infection-related death, while maintaining the relative dose intensity (RDI) of chemotherapy and, therefore, the effectiveness of cancer treatment (Cornes et al. 2020).

The National Comprehensive Cancer Network (NCCN) clinical practice guidelines support the use of G-CSF in high-risk patients, that is patients with a risk of developing chemotherapy-related FN of 20% or greater. Similarly, G-CSF use is recommended when the risk is estimated to be between 10% and 20% (intermediate risk) in a patient with additional risk factors. Granulocyte colony-stimulating factor is not recommended in low-risk patients (a risk of less than 10%) (Jiménez Nieves et al. 2022).

Despite the available guidelines for G-CSF administration, literature reports the inconsistent use of G-CSF in clinical settings. Several studies reported variations in the use of G-CSF. These studies have demonstrated the underutilisation of G-CSF in patients receiving chemotherapy treatment associated with a high risk of developing FN and inappropriate prescription of G-CSF in low-risk patients (Barnes, Pathak & Schwartzberg 2014; Hawkins et al. 2020; Okunaka et al. 2021; Wright et al. 2013).

This study was aimed at evaluating the prescribing patterns of G-CSF in the primary prophylaxis of patients receiving chemotherapy at Dr George Mukhari Academic Hospital (DGMAH) as compared to recognised guidelines. Practice guidelines may be useful in producing better care and decreasing costs and length of hospital stay.

## Research methods and design

### Study design and setting

This was a quantitative, descriptive, retrospective study and patient data were retrieved through patient files. The study was conducted at the haematology oncology wards of DGMAH over a period of 7 months from 01 January 2018 to 31 July 2018.

### Study population and sample

The study population consisted of all patients admitted between 01 January 2018 and 31 July 2018 and who received one or more cycles of myelosuppressive chemotherapy. Purposive systematic sampling as per inclusion criteria was used to identify the files of adult patients treated at the hospital during the study period. The selected files were reviewed to extract the necessary data. Inclusion criteria were as follows: patients over the age of 18 years who had received two or more cycles of myelosuppressive chemotherapy and who developed neutropaenia.

### Data collection and analysis

The data collection tool was developed based on recommendations from the American Society of Clinical Oncology (ASCO) and European Organisation for Research and Treatment of Cancer (EORTC) treatment guidelines and contained the following variables: patient demographics, clinical data, prescribed treatment and effects of neutropaenia on chemotherapy treatment.

Demographic details included age, ethnic group, gender, weight and height of the patient. This information provided an overview of the patient’s background. Clinical data included patient’s medical history, primary diagnosis and full blood count results. Data obtained from this section provided information on disease status and possible outcomes. The treatment prescription was used to determine if G-CSF was prescribed as primary or secondary prophylaxis and if supportive treatment was offered as per guideline recommendations.

The effects of neutropaenia on chemotherapy dosing were inferred from the time to initiation of therapy, chemotherapy dose reduction or prolonged patient hospitalisation.

All data were captured on MS Excel™ spreadsheets and reviewed by a colleague for accuracy and completeness. The necessary corrections were made prior to data analysis, through consultation with a statistician. All statistical analyses were performed on SAS (SAS Institute INC, Carey NC, USA), Release 9.4, running under Microsoft Windows for a personal computer.

## Ethical considerations

Patient files were numbered to maintain confidentiality and no individual data were presented. Data collection commenced only after receiving approval from the Sefako Makgatho University Research Ethics Committee (SMUREC/P/130/2017:PG) and also after receiving approved consent from the Chief Executive Officer of the hospital and all healthcare professionals involved in the management of oncology patients.

The study was retrospective in nature and patient data were obtained from treatment records; therefore, patient consent was not required. Data were handled confidentially and anonymously and patient identifiers were excluded from the data collection tool.

## Results

A total of 128 adult patient files were screened for eligibility, of which 57 met the inclusion criteria and were included in the final analysis.

### Demographics

Of the 57 cases, 42 (74%) were males and 15 (26%) females. Fifty-four (95%) patients were black ethnicity and three (5%) from the white ethnic group. Patient weight ranged from 39 to 153 kg with a mean of 79.70 kg. The mean age was 40.86 (± 11.95) years with a range of 19–71 years.

## Clinical data

### Types of malignancies

Table 1 illustrates the types of malignancies that were diagnosed and treated during this period. The diagnoses were recorded as is from the patient files and included: Hodgkins lymphoma (HL) 10 patients (17.54%), plasmablastic lymphoma (PBL) seven patients (12.28%), T-cell acute lymphoblastic leukaemia (T-ALL) seven patients (12.28%), B-cell acute lymphoblastic lymphoma (B-ALL) seven patients (12.8%), non-Hodgkin lymphoma (NHL) seven patients (12.8%), diffuse large B-cell lymphoma (DLBCL) six patients (10.53%), multiple myeloma (MM) five patients (8.77%), nodular sclerosing Hodgkin lymphoma (NSHL) four patients (7.02%), and Burkitt lymphoma (BL) four patients (7.02%).

**TABLE 1:** Types of malignancies.

Type	*n* (57)	Percentage (100%)
Hodgkin lymphoma	10	17.54
B-cell acute lymphoblastic lymphoma	7	12.28
Non-Hodgkin lymphoma	7	12.28
Plasmablastic lymphoma	7	12.28
T-cell acute lymphoblastic leukaemia	7	12.28
Diffuse large B-cell lymphoma	6	10.53
Multiple myeloma	5	8.77
Burkitt lymphoma	4	7.02
Nodular sclerosing Hodgkin lymphoma	4	7.02

#### Co-morbid conditions

Patients with co-morbid diseases and those treated for other complications were grouped according to major systems (Table 2). The majority were HIV positive (*n* = 18; 31.58%). Comorbidities included pulmonary complications (*n* = 10; 17.58%), cardiovascular (*n* = 8; 14.04%) and CNS (*n* = 1; 1.75%). Twenty (35.09%) patients had no concomitant or other co-morbid diseases.

**TABLE 2:** Co-morbid conditions.

Co-morbidity	*n* (57)	Percentage (100%)
HIV	18	31.58
Pulmonary	10	17.54
Cardiovascular	8	14.04
CNS	1	1.75
None	20	35.09

CNS, Central nervous system.

### Types of chemotherapy regimens used

**FIGURE 1:**
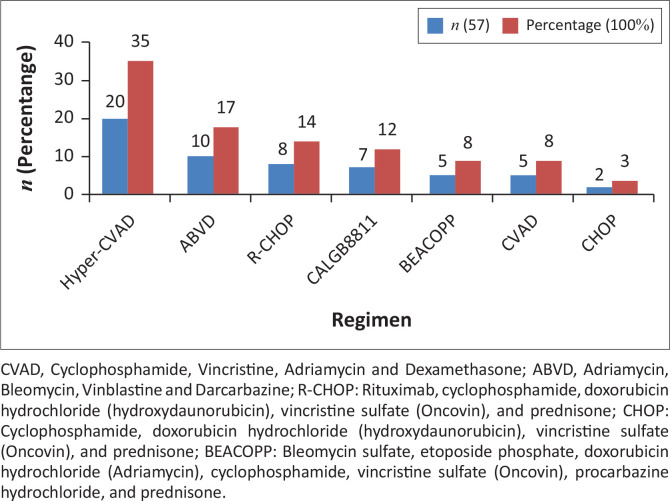
Types of chemotherapy regimens used.

In this study, systemic chemotherapy was given either palliatively or with curative intent. Figure 1 depicts the various regimens received by patients who presented with haematological malignancies at DGMAH during the study period. Doses of chemotherapy agents were based on the patient’s weight, height and body surface area. The two most used chemotherapeutic regimens were Hyper-CVAD (Cyclophosphamide, Vincristine, Adriamycin and Dexamethasone) (35.09%) and ABVD (Adriamycin, Bleomycin, Vinblastine and Darcarbazine) (17.54%).

### Use of colony-stimulating factor

Table 3 demonstrates the prescribing patterns (dose and duration) of G-CSF in accordance with known guidelines. The recommended dose for patients weighing less than 75 kg and more than 75 kg was 30 MU and 48 MU, respectively. Twenty-five patients (43.86%) did not receive G-CSF doses according to the recommended guidelines. In 30 patients (52.63%), the duration of treatment was not in accordance with recommended guidelines where treatment continued after the absolute neutrophil count (ANC) surpassed 10 000/mm^3^ and chemotherapy-induced ANC nadir has occurred. Therefore, overuse of G-CSF was observed in this study.

**TABLE 3:** Usage of granulocyte colony-stimulating factor.

Usage	*n* (57)	Percentage (%)
G-CSF	57	100.00
Dose according to guideline	32	52.14
Dose not according to guideline	25	43.86
Duration according to guideline	27	47.37
Duration not according to guideline	30	52.63

G-CSF, granulocyte colony-stimulating factors.

### Parameters considered when using granulocyte colony-stimulating factors

Forty patients (70.17%) received G-CSF as primary prophylaxis during their first cycle of chemotherapy and in 17 patients (29.82%), G-CSF was prescribed as part of secondary prophylaxis. Thirteen (22.81%) patients on G-CSF therapy presented with advanced stage disease as shown in Table 4.

**TABLE 4:** Parameters considered when using granulocyte colony-stimulating factor.

Parameter	*n* (57)	Percentage (100%)
Primary prophylaxis	40	70.17
Age > 65 years	5	8.77
Advanced disease stage	13	22.81
Active infections or increased risk of infections	27	47.37
Secondary prophylaxis: Prophylactic use for subsequent cycles after initial episode(s) of severe and/or febrile neutropaenia in chemotherapy regimens where dose reduction or delays would compromise outcomes	17	29.82
Supportive management of severe neutropaenic sepsis or prolonged neutropaenia.	12	21.05

### Effects of neutropaenia on chemotherapy delivery

**FIGURE 2:**
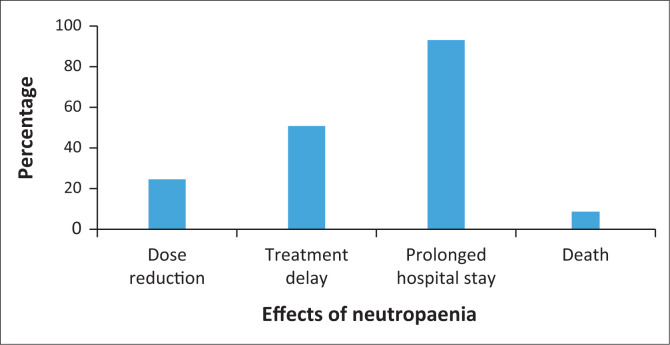
Effects of neutropaenia on chemotherapy.

Dose reduction was initiated in 14 patients (24.56%). Twenty-nine patients (50.88%) experienced treatment delays. Almost all the patients (2.98%) required prolonged hospitalisation as seen in Figure 2. There were five deaths (8.77%) recorded. The cause of death was not clearly documented and could not be attributed to neutropaenia.

## Discussion of results

The burden of HL (most prevalent cancer type in this study) is dependent on gender, age and geographical location (Zhou et al. 2019). According to the Herbst (2021), 356 males and 293 females were diagnosed with HL in South Africa during 2017 (Herbst 2021). The association between the male gender and moderate or severe CIN may also be due to the preponderance of males (74%) to females (26%) in the study. The racial distribution in this study could be attributed to referral patterns given geographical location of this patient population. The study was conducted in a semi-rural area with patients residing mainly in local townships and rural areas. Hodgkins lymphoma is associated with a bimodal distribution usually between the ages of 15 and 40 years and over the age of 55 years (Kaseb & Babiker 2022). In this study, the mean patient age was 40 years.

With the introduction of highly active antiretroviral therapy (HAART) and the expected improved life expectancy, malignancies have become the leading cause of disease and death in people with HIV. Although HL is not considered an AIDS-defining disease, the incidence of HL in HIV-infected individuals is higher compared to the general population (Moahi et al. 2022). More than 28% of HIV-related deaths are ascribed to malignant tumours and AIDS-associated lymphomas are found in more than 40% of HIV-infected individuals (Berhan, Bayleyegn & Getaneh 2022). Hodgkins lymphoma is the most common cancer treated at Dr George Mukhari Academic Hospital (17.54%). These results correlate with those from other South Africa (SA) tertiary hospitals, where the prevalence of HL accounted for 7% – 17% of all lymphomas (Alli & Meer 2017; Naidoo et al. 2018). However, epidemiological studies of HL in other African countries demonstrated a prevalence of 7% – 12.5% of all reported cases of lymphoma (Westmoreland et al. 2017). HIV-associated lymphoma (HAL) is a common malignancy in South Africa (Wang, Jun & Yao, 2022), although there are limited data from South Africa on HL (Rapiti et al. 2022). This study found HIV a comorbid disease in most patients treated for lymphomas which is in line with published literature (Moahi et al. 2022).

Pulmonary complications (17.54%) were common in this study. Vadde and Pastores (2016) found that pulmonary complications occurred in 10% – 20% of patients with acute leukaemia or lymphoma and in nearly 50% of patients with neutropaenia (Vadde & Pastores 2016). Cardiovascular (CVS)-related disease and treatment were noted in 14.04% of patients. It was unclear if chemotherapy was the causative factor or whether CVS disease was due to factors unrelated to chemotherapy. Mozos and colleagues (2017) demonstrated that CVS diseases are often associated with chemotherapy. This includes hypertension, myocardial infarction, stroke and peripheral vascular disease.

Various chemotherapy regimens were used for the treatment of haematological malignancy at DGMAH with Hyper-CVAD found to be the most used regimen. Jalaeikhoo et al. (2018) also reported that the Hyper-CVAD regimen is widely used. However, Verburgh and Antel (2019) recommended that Hyper-CVAD should only be prescribed for high-grade or highly aggressive lymphoma.

In this patient cohort, almost 80% of patients presented with grade 1 neutropaenia. Sapkota and colleagues (2020), on the other hand, evaluated 203 patients and found that 163 (80.29%) patients suffered from neutropaenia, with only 14 (6.89%) cases of grade 1 neutropaenia, while 149 (73.39%) patients suffered severe neutropaenia. This could be due to the masking of neutropaenia from prior G-CSF therapy. This may have had an effect on subsequent neutrophil counts and altered the level of bone marrow suppression seen as neutrophil recovery is improved.

The prevention of neutropaenia and FN in patients receiving chemotherapy for the treatment of malignant disease is achieved with the administration of haematopoietic growth factors. All the patients who were enrolled in the study received G-CSF. Duration of treatment with G-CSF was not according to guidelines in more than 50% of the patients. The guidelines recommend that G-CSF should be continued until recovery of the post-nadir ANC to normal or near-normal levels of 2–3 × 10^9^/L (Crawford et al. 2017). In this study, G-CSF was given for longer periods than recommended and, therefore, overuse of G-CSF was observed. Commercially, the drug is available in vials as two dosage strengths, that is 300 µg and 480 µg. Because of this limitation in dosage formulations, it was found that 43.86% of patients received an incorrect dose and were given 300 µg or 480 µg regardless of patient weight. The dose formulations of pre-filled syringes limit dose adjustments and contribute to over- or under-dosing as opposed to weight-specific dosing. According to Zullo et al. (2019), several studies reported that the inconsistency of G-CSF administration is attributed to under- and over-utilisation.

The desired indications for G-CSF use in this study were defined by EORTC and ASCO guidelines as South Africa does not have published guidelines. Guideline recommendations indicate that primary prophylaxis with G-CSF is administered 24–72 h immediately after cycle 1 of chemotherapy. In this study, the authors found that 70.17% of patients were given G-CSF as primary prophylaxis in their first cycle of chemotherapy which is comparable with a study by Zekri and colleagues (2018), where 69.9% of patients received primary prophylaxis G-CSF after the first cycle of chemotherapy.

One approach to manage FN is to delay or reduce the dose of chemotherapy, which may have deleterious consequences for patients, including increased mortality (Pettengell et al. 2009). In this study, dose reduction was observed in 24.56% of patients. These findings are similar to the findings of the ChemoInsight Project, a large ongoing retrospective analysis of patient records in the United States. This project found that dose delays were implemented in 43.1% of 20,106 cases, while 25.7% required dose reductions (Leonard et al. 2003).

Half of the evaluated cases at DGMAH (50.88%) experienced treatment delays. Almost all the patients experienced prolonged hospitalisation (92.98%). Nattamol and colleagues (2015) reported a 55.9% prolonged hospitalisation rate. This could be attributed to an increased prevalence of comorbidities in this study. Prolonged neutropaenia leads to prolonged hospitalisation and increased risk of hospital-acquired infections (Krishnamani et al. 2017).

## Limitations

The study represents a small proportion of patients treated at tertiary South African institutions. The study was further hampered by a small sample size and a limited cross-sectional review. Data were obtained from patient files where information was often incomplete or difficult to retrieve.

## Recommendations

Primary prophylaxis with G-CSF should be started earlier in high-risk cases to reduce the risk of serious complications in cancer patients. This will negate dose adjustments or treatment interruptions. Flexible G-CSF dosing would minimise over- or under-prescribing as doses would be calculated according to weight. The pharmacist has an important role to play in gatekeeping and ensuring safe, effective prescribing and adherence to local guidelines.

## Conclusion

Neutropaenic events may negatively influence chemotherapy dosing. Granulocyte colony-stimulating factors are recommended as primary prophylaxis to reduce the risk of FN and to maintain the desired dose intensity of chemotherapy to ensure the effective treatment of cancer. This study demonstrated unsatisfactory compliance to guidelines and overuse of G-CSF. Although the indications were appropriate, over-dosage or prolonged duration of treatment beyond the desired nadir was a common occurrence.
